# Mixed-Curve Model for Evaluating the Carbonation Depth of Concrete at Different Ages

**DOI:** 10.3390/ma17194710

**Published:** 2024-09-25

**Authors:** Xinhao Wang, Qiuwei Yang, Hongfei Cao, Fengjiang Qin

**Affiliations:** 1 School of Civil and Transportation Engineering, Ningbo University of Technology, Ningbo 315211, China; wangxinhao0709@163.com (X.W.); caohongfei@nbut.edu.cn (H.C.); 2 Engineering Research Center of Industrial Construction in Civil Engineering of Zhejiang, Ningbo University of Technology, Ningbo 315211, China; 3 Key Laboratory of New Technology for Construction of Cities in Mountain Area, Ministry of Education, Chongqing University, Chongqing 400045, China; 4 School of Civil Engineering, Chongqing University, Chongqing 400045, China

**Keywords:** concrete, elastic modulus, compressive strength, two-parameter curve model, three-parameter curve model

## Abstract

To accurately quantify the variation in concrete carbonation depth, selecting an appropriate mathematical curve model is crucial. Currently prevalent models, such as the Fick model and exponential models, confront limitations in prediction accuracy and range of application. Given that a single curve model struggles to precisely describe the pattern of concrete carbonation, this work introduces a mixed-curve-based prediction model for carbonation depth, effectively integrating the Fick model with a hyperbolic model. Compared to the Fick model, the additional term in the mixed-curve model can be viewed as a reasonable correction to better adapt to the complex and varied conditions of concrete carbonation. This hybrid model transcends the limitations of individual models, enhancing fitting precision and broadening the scope of applicability. The new model boasts a concise structure with only two fitting parameters, facilitating ease of application. To validate its superiority, rigorous comparisons were conducted between the proposed model and existing ones, leveraging experimental data from 10 distinct concrete carbonation scenarios. By comparing the average error, standard deviation, and coefficient of determination across these cases, the new model demonstrates a clear advantage over the Fick model and the exponential model. In terms of fitting errors, the average error and standard deviation of the new model are notably lower than those of the other two models. In terms of the coefficient of determination, the values achieved by the new model in all examples are closer to 1 than those of both the Fick model and the exponential model, underscoring the new model’s superior fitting quality and remarkable stability. This research indicates that the combined model presented in this paper holds promising prospects for widespread application in predicting concrete carbonation depth.

## 1. Introduction

**Figure 1:**
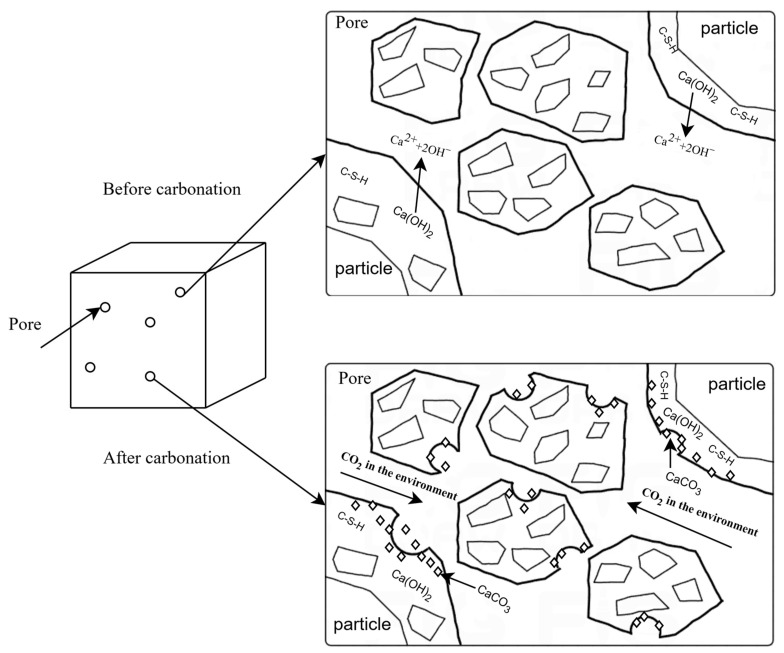
Carbonation reaction mechanism.

Concrete carbonation occurs when atmospheric CO_2_ permeates concrete pores and cracks, chemically reacting with alkaline components to decrease alkalinity. Key reactions are detailed in Equations (1)–(4) [1], with pore alterations depicted in Figure 1 [2]. Note that Equations (2)–(4) are verified only for high CO_2_ concentrations (>5%). This process lowers pH near reinforcing steel, accelerating corrosion, damaging structures, and reducing their lifespan [3,4]. The assessment of concrete carbonation depth is of great significance in ensuring structural safety and estimating service life [5,6,7,8,9,10,11,12].

(1) $$\mathrm{Ca}{\left(\mathrm{OH}\right)}_{2}+{\mathrm{CO}}_{2}\overset{H_{2}O}{\to }{\mathrm{CaCO}}_{3}+H_{2}O$$

(2) $$\left(3\mathrm{CaO}\cdot {\mathrm{SiO}}_{2}\cdot 3H_{2}O)+3{\mathrm{CO}}_{2}\to (3{\mathrm{CaCO}}_{3}\cdot 2{\mathrm{SiO}}_{2}\cdot 3H_{2}O\right)$$

(3) $$3\mathrm{CaO}\cdot {\mathrm{SiO}}_{2}+3{\mathrm{CO}}_{2}+\gamma H_{2}O\to {\mathrm{SiO}}_{2}\cdot \gamma H_{2}O+3{\mathrm{CaCO}}_{3}$$

(4) $$2\mathrm{CaO}\cdot {\mathrm{SiO}}_{2}+3{\mathrm{CO}}_{2}+\gamma H_{2}O\to {\mathrm{SiO}}_{2}\cdot \gamma H_{2}O+2{\mathrm{CaCO}}_{2}$$

Currently, there are two main methods for evaluating concrete carbonation depth (CD): machine learning techniques and mathematical curve fitting. Among the machine learning approaches, various models are constructed, including artificial neural networks (ANN), support vector machines (SVM), and decision trees (DT), which are subsequently trained on extensive datasets to accurately evaluate the CD of concrete. This reliance on large data sets allows for more nuanced and precise assessments compared to traditional methods. Majlesi et al. [13] trained an ANN model for predicting the CD of reinforced concrete under different natural and environmental conditions. Tran [14] devised an ANN model to forecast CD, utilizing 300 experimental data points for validation. Felix et al. [2] employed an artificial neural network (ANN) coupled with a backpropagation algorithm for the purpose of predicting the CD of concrete that incorporates fly ash additives. Concha [15] created a new CD prediction model using ANN, analyzing 445 experimental data points for enhanced accuracy. Akpinar and Uwanuakwa [16] refined carbonation simulation and prediction accuracy with an ANN model utilizing a scale conjugate gradient (SCG) function. Zhang et al. [17] introduced a hybrid prediction framework merging the least squares support vector machine (LSSVM) with a metaheuristic algorithm to precisely forecast the CD of fly ash-blended concrete. Huang et al. [18] compared ANN, radial product function neural networks (RBF), random forests (RF), and SVM for CD prediction under varying conditions. Çevik et al. [19] reviewed SVM’s applications in structural engineering, citing case studies. Li et al. [20] applied SVM to forecast concrete carbonation, discovering its predictive accuracy surpassed Back Propagation (BP) neural networks. Ruan [21] introduced the Support Vector Regression (SVR) to predict concrete CD, validated with real case data of CD. Londhe et al. [22] introduced Model Tree (MT), RF, and Multi Gene Genetic Programming (MGGP) for predicting concrete carbonation coefficients. Wang et al. [23] comprehensively evaluated eight ML algorithms (DT, RF, AdaBoost, CatBoost, XGBoost, K-Nearest Neighbors (KNN), SVR, and Multilayer Perceptron (MLP)) for predicting CD in concrete with solid waste. Amirhossein et al. [24] crafted artificial bee colony expression programming (ABCEP) models, rigorously analyzed, and compared the best ones with previous models. Lee et al. [25] utilized a state-of-the-art deep learning model to forecast concrete carbonation, outperforming the Architectural Institute Japanese (AIJ) Model and the Finite Element Method (FEM) analysis. Kumar et al. [26] introduced an adaptive neural fuzzy inference system (ANFIS) for estimating CD in fly ash concrete.

The model evaluated by combining mathematical curves mainly considers the influence of concrete composition on the CD of concrete. The fundamental concept behind this approach involves establishing the functional relationship between the mechanical properties of concrete and the various influencing factors based on initial experimental data. This information is then leveraged to apply data-fitting techniques, allowing for the calculation of concrete’s mechanical properties under a range of different influencing conditions. Through this process, a clearer understanding of how varying factors affect performance can be achieved, leading to more accurate predictions and analyses. At present, the main models include the evaluation model based on the water–cement ratio [27,28], the evaluation model based on concrete compressive strength [29,30], and the evaluation model considering multiple parameters [31,32]. The original model was based on Fick’s diffusion law to predict the depth of carbonation, and the original model was called the Fick model [33]. Other scholars have conducted in-depth research based on the Fick model and extended this model. For example, Possan et al. [34] proposed a mathematical model for estimating the CD of concrete and predicting the service life of concrete structures under carbon dioxide, which was validated using multiple sets of data. The results indicate that the model has the potential to predict the CD of concrete under boundary conditions that guide its development. Ekolu [35] proposed a mathematical model for predicting the effects of concrete composition and environmental factors on natural carbonation. Multiple datasets were used for model development and calibration, and the proposed model was compared with the fib model. The results indicate that the proposed model is relatively accurate. Liang and Lin [36] developed a new one-dimensional mathematical model. A one-dimensional linear partial differential equation was derived based on the principle of mass balance and convection dispersion equation, and an analytical solution was found through mathematical methods. Various parameters determine the numerical results of concrete carbonation. The numerical results indicate the practical application of the model. These results indicate that the proposed model can describe the carbonation process of concrete chemically and physically. The advantage of mathematical curve models lies in their ability to directly establish functional relationships between concrete CD and a multitude of influencing variables, such as the water-to-cement proportion, material temperature, pressure, and the chemical makeup of the constituents [2].

In general, while existing evaluation models are capable of predicting the mechanical properties of concrete to some degree, they exhibit certain limitations in specific areas. The drawbacks of machine learning-based evaluation models can primarily be categorized into two main issues: first, these models require a training phase before they can be effectively utilized for assessments; second, the complexity of the parameters considered by machine learning evaluation models can make their application in practical engineering challenging. This complexity can hinder their ease of use in real-world scenarios. The limitations of current mathematical curve models mainly lie in their limited applicability. For example, multi-parameter models must consider multiple factors and require a large amount of experimental data for fitting, which can lead to defects such as difficulty in obtaining variable data. To overcome the problems of insufficient early data and difficulty obtaining various variables in practical engineering, this study aims to develop a new dual-parameter curve model by integrating the advantages of existing curve models. In comparison to pre-existing curve formulations, the present model offers improved precision in assessing the interplay between the CD and the temporal evolution of concrete carbonation. Drawing upon the outcomes of this model’s evaluations, a more nuanced comprehension of the CD within the component is achievable, thereby facilitating the anticipation and expeditious adoption of preventive actions. Therefore, this article first analyzes the advantages and disadvantages of the original Fick model and exponential model and then proposes a new digital curve model that overcomes the shortcomings of existing curve models. The improvement of the new model is mainly reflected in two aspects: firstly, the mathematical formula of the new model is concise, only considering the fitting of two parameters, which is convenient for practical engineering applications; second, the curve used in the new model has been improved based on the Fick model, resulting in higher fitting accuracy than existing models. This article mainly studies the application of a new model in the assessment of CD in concrete. This study engages in a comparative analysis utilizing ten sets of experimental data sourced from the established literature, aiming to evaluate the computational accuracy of a new model against existing ones. The objective is to highlight the advancements and improved performance of the new model over its predecessors.

## 2. Mathematical Models for Predicting the Carbonation Depth of Concrete

### 2.1. Analysis of Existing Models

The existing evaluation models that describe the relationship between the age of concrete and CD primarily consist of power function, exponential, and logarithmic models. The foundational power function model is the Fick model, and the corresponding curve equation is as follows [33]:

(5) $$z(t)=a\sqrt{t}$$

Among them, z(t) represents the quantified CD of the concrete at the specific time denoted by t; a denotes an unknown non-negative parameter that must be determined using experimental data. As indicated in Equation (5), a key advantage of the Fick model is that it requires fewer parameters for fitting, which facilitates its application in practical engineering scenarios. However, the Fick model also has its limitations. The primary drawback is that it incorporates only one parameter for fitting; thus, when dealing with experimental data that exhibit considerable variability, the fitting accuracy may be reduced, leading to substantial errors.

The curve equation of the exponential model is expressed as [37]:

(6) $$z(t)=a+b\cdot e^{-t/c}$$

In Equation (6), a, b, and c are the three fitting parameters that need to be obtained through curve fitting based on experimental data. The advantage of this model is that the model function is a monotonic function, which is consistent with the increasing trend of concrete CD over time in practical engineering. And when t→0, z(t) tends to converge. However, due to the need to fit three parameters in this model, more experimental data are required for fitting, making it less convenient for practical engineering applications.

### 2.2. The Proposed Curve Model

Due to the numerous factors influencing concrete carbonation, such as concrete strength and porosity, it is challenging to precisely capture the variation pattern of concrete carbonation depth using a single curve model. This is also the underlying reason for the poor fitting accuracy of traditional models. A promising approach is to combine various curves into a mixed model to adapt to the complexity and variability of concrete carbonation conditions. In view of this, this work proposes a new mixed-curve model, which improves the accuracy of describing the variation in concrete carbonation depth over time in practical engineering applications. The mathematical expression of this mixed-curve model is provided below:

(7) $$z(t)=a\sqrt{t}+b\cdot \frac{\sqrt{t}}{\sqrt{t}+1}$$

In Equation (7), t and z(t) represent the time and the corresponding concrete carbonation depth at that time, consistent with the definitions provided in previous equations. The two unknown parameters a and b are determined by fitting experimental data. Comparing Equation (7) with Equation (5), it can be seen that this new model is a hybrid of the Fick model and the hyperbolic model, where the additional term can be regarded as a reasonable modification to the Fick model to adapt to different concrete carbonation conditions. The newly added term $\frac{\sqrt{t}}{\sqrt{t}+1}$ exhibits the following characteristics: when t = 0, $\frac{\sqrt{t}}{\sqrt{t}+1}$ = 0, and as t approaches infinity, $\frac{\sqrt{t}}{\sqrt{t}+1}$ approaches 1. This indicates that the added term $\frac{\sqrt{t}}{\sqrt{t}+1}$ is a bounded function that can, to a certain extent, compensate for the shortcomings of the Fick model in adapting to complex and variable carbonation scenarios, thereby enhancing the fitting accuracy. Compared to other mathematical curve models that involve multiple influencing parameters, the proposed combined curve model boasts a simple and clear structure, containing only two unknown fitting parameters, making it more suitable for engineering applications.

### 2.3. Solving Process of Fitting Parameters

In this section, we will further discuss the method for calculating the fitting parameters in the previously proposed mathematical curve model. To begin, we will create a linear equation system that relates CD to age, using the actual data obtained from experiments. Subsequently, we will derive the corresponding fitting parameters. Through the employment of diverse collections of experimental data, the linear system pertaining to Equation (7) can be formulated in the manner depicted below:

(8) y=C·x

(9) $$y=\left\{\begin{array}{c} z(t_{1})\\ \vdots \\ z(t_{n}) \end{array}\right\},$$

(10) $$x=\left\{\begin{array}{c} a\\ b \end{array}\right\}$$

(11) $$C=\left[\begin{array}{cc} \sqrt{t_{1}} & \frac{\sqrt{t_{1}}}{\sqrt{t_{1}}+1}\\ \vdots & \vdots \\ \sqrt{t_{n}} & \frac{\sqrt{t_{n}}}{\sqrt{t_{n}}+1} \end{array}\right]$$

where $z(t_{n})$ denotes the CD of the concrete specimen measured at time $t_{n}$. It is evident that both the coefficient matrices and vectors in Equation (8) can be derived from the experimental data. Consequently, the least squares method can be employed to determine the vector of undetermined fitting coefficients x. Thus, Equation (8) can be reformulated as follows:

(12) $$C^{T}y=(C^{T}C)\cdot x$$

Pursuant to Equation (11), the representation of the solution for x is formulated as

(13) $$\hat{x}={\left(C^{T}C\right)}^{-1}C^{T}y$$

**Figure 2:**
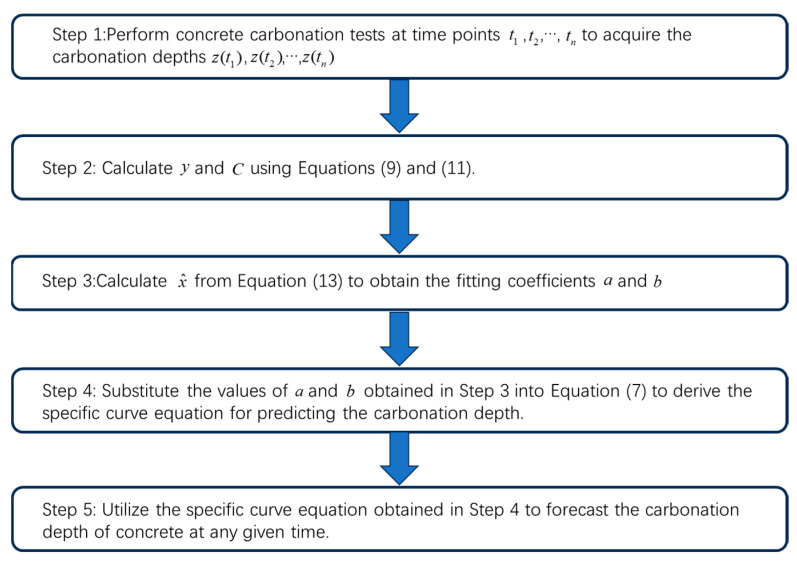
Schematic diagram of the process for predicting the depth of carbonation in concrete.

The computed value of $\hat{x}$ represents the least squares estimation of x. After obtaining the fitting parameters using the above formulas, the specific curve model is correspondingly established for predicting the carbonation depth. Different fitting parameters can be obtained by employing the aforementioned curve-fitting method for different concrete strengths, porosities, and other relevant factors. These fitting parameters, in turn, enable the generation of unique prediction curves for carbonation depth that precisely correspond to each specific concrete carbonation condition. In other words, the proposed model precisely adapts to changes in the properties of concrete materials and environmental conditions by adjusting the fitting parameters. This is a common characteristic shared by predictive models of this kind, which is to accommodate changes in the properties of the research object by adjusting the built-in parameters of the model. Figure 2 provides a more intuitive illustration of the operational process of the proposed model for predicting the carbonation depth of concrete.

To assess the accuracy of these model predictions, we can compare the predicted values against the experimental values. The quantitative divergence between the predicted outcomes and the experimental measurements is defined as follows:

(14) $$\left\{\delta \right\}=\left|C\cdot \hat{x}-y\right|$$

where $\left\{\delta \right\}={\left(\delta_{1},\cdots ,\delta_{n}\right)}^{T}$ is referred to as the residual vector. In particular, $\delta_{i}$ represents the residual error between the ith experimental data point and its corresponding predicted value. The average and standard deviation of these residual errors can be computed using the following methods:

(15) $$\bar{\delta }=\frac{\delta_{1}+\cdots +\delta_{n}}{n}$$

(16) $$\sigma =\sqrt{\frac{\sum_{i=1}^{n}{\left(\delta_{i}-\bar{\delta }\right)}^{2}}{n}}$$

In this context, $\bar{\delta }$ denotes the average of the residual errors, while σ represents the corresponding standard deviation. A lower value of $\bar{\delta }$ and σ indicates a greater accuracy in the curve model fitting.

To evaluate the model’s predictive capability, the coefficient of determination ($R^{2}$) is calculated. This metric assesses the degree of closeness between the predicted and experimental values, thereby providing insights into the model’s effectiveness in estimating CD. The formula for calculating $R^{2}$ is as follows:

(17) $$R^{2}=1-\frac{\sum_{i=1}^{n}{\left(y_{e}-y_{p}\right)}^{2}}{\sum_{i=1}^{n}{\left(y_{e}-{\bar{y}}_{e}\right)}^{2}}$$

where $y_{e}$ = experimental value; ${\bar{y}}_{e}$ = mean of experimental values; $y_{p}$ = predicted value, and n = total number of observations

## 3. Model Verification and Comparison via Concrete Experiment Data

We validated the results of the new evaluation model proposed in this paper by collecting experimental data from references [38,39,40,41,42,43,44,45,46,47] (Case 1–10). The fitting results are compared with the existing Fick and exponential models to demonstrate the superiority of the new model.

Case 1: In citation [38], some fly ash concrete specimens measuring 100 mm × 100 mm × 400 mm were cast and subjected to standard curing for 28 days. Initially, the CD of the concrete was measured after 0 days of curing. Subsequently, the fly ash concrete underwent rapid carbonation treatment following the initial measurement. The concentration of carbon dioxide for rapid carbonation is set to 20% + 3%; the temperature is set to 20 °C + 2 °C, and the relative humidity is set to 70% ± 5%. The CD was measured after three days, seven days, 14 days, and 28 days of rapid carbonation treatment. The average value of these measurements for each time point was considered the CD. Table 1 shows the material composition of the fly ash concrete, while Table 2 presents the CD data for each time point.

**Figure 3:**
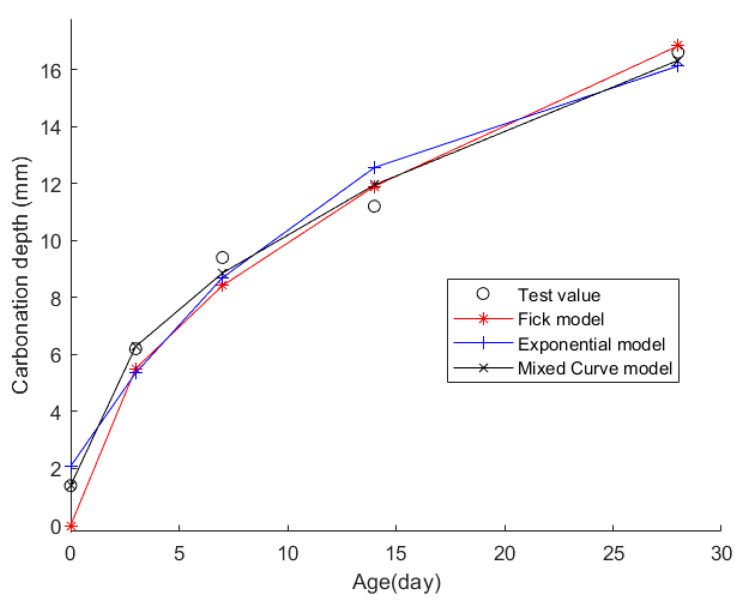
The fitting results of Case 1.

The obtained data were then fitted into the Fick, exponential, and mixed models for analysis. The resulting fitting curves can be seen in Figure 3. Table 3 displays the models’ average fitting error, standard deviation, and coefficient of determination ($R^{2}$). The specific fitting equations of the three models are as follows:

(18) $$z(t)=3.1799\sqrt{t}$$

(19) $$z(t)=17.9559-15.8783\cdot e^{-t/12.9828}$$

(20) $$z(t)=2.8194\sqrt{t}+1.7914\cdot \frac{\sqrt{t}}{\sqrt{t}+1}$$

Case 2: In citation [39], some standard-sized lime powder concrete test blocks were poured and cured under standard conditions for seven days before being cured under natural conditions. Then, concrete CD at 14, 28, 60, and 90 days of curing was measured. The materials used include the following: Class II fly ash with a fineness of 18.0% and a water requirement of 101%; S105 grade ground granulated blast-furnace slag; limestone powder with 20% residue on a 45 μm sieve and a flowability ratio of 101%; Polycarboxylate superplasticizer; medium sand with a fineness modulus of 2.67 and an apparent density of 2630 kg/m^3^; granite gravel with a particle size ranging from 5 to 25 mm and an apparent density of 2670 kg/m^3^; and P·O 42.5 grade cement. Table 4 shows the material composition of concrete, while Table 5 displays the CD data at each time point.

**Figure 4:**
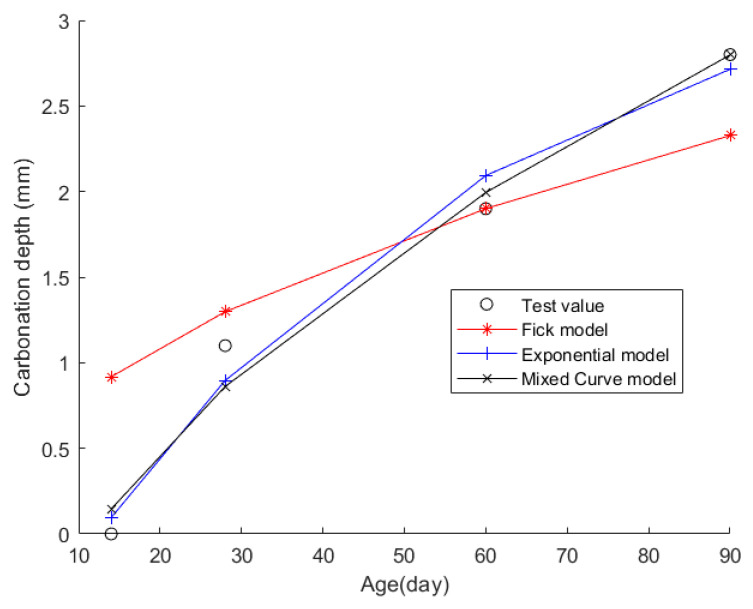
The fitting results of Case 2.

Then, the obtained data were put into the Fick, exponential, and mixed models for analyses. Figure 4 shows the final fitting curve. Table 6 shows the model’s average fitting error, standard deviation, and coefficient of determination ($R^{2}$). The specific fitting equations for the three models are as follows:

(21) $$z(t)=0.2453\sqrt{t}$$

(22) $$z(t)=3.5205-4.4714\cdot e^{-t/52.53}$$

(23) $$z(t)=0.4617\sqrt{t}-1.8014\cdot \frac{\sqrt{t}}{\sqrt{t}+1}$$

Case 3: In citation [40], several C30 concrete cube samples measuring 100 mm on each side were cast and underwent conventional curing procedures. The CD of the concrete was measured after 3, 7, 14, and 28 days of the curing process. This design employs 42.5 grade ordinary Portland cement as the primary binding material. For fine aggregate, high-quality river sand with an apparent density of 2640 kg/m^3^, a fineness modulus of 2.9, and a low mud content of only 0.5% is selected, belonging to the Grade II gradation zone with excellent gradation. As for coarse aggregate, two types of continuously graded crushed stones are used: the first is granite crushed stone with an apparent density of 2800 kg/m^3^, a crushing value of 6.0%, a mud content of 1.0%, and a particle size range of 2.5 to 20.0 mm; the second is limestone crushed stone with an apparent density of 2740 kg/m^3^, a crushing value of 7.9%, a similar mud content of 1.0%, and an extended particle size range of 2.5 to 40.0 mm. Additionally, Grade I fly ash is introduced, featuring a fineness of 8.4, a moisture content of 0.3%, and a water requirement of 95%. The material composition of the concrete is detailed in Table 7, with Table 8 presenting the CD values recorded at the specified time intervals.

**Figure 5:**
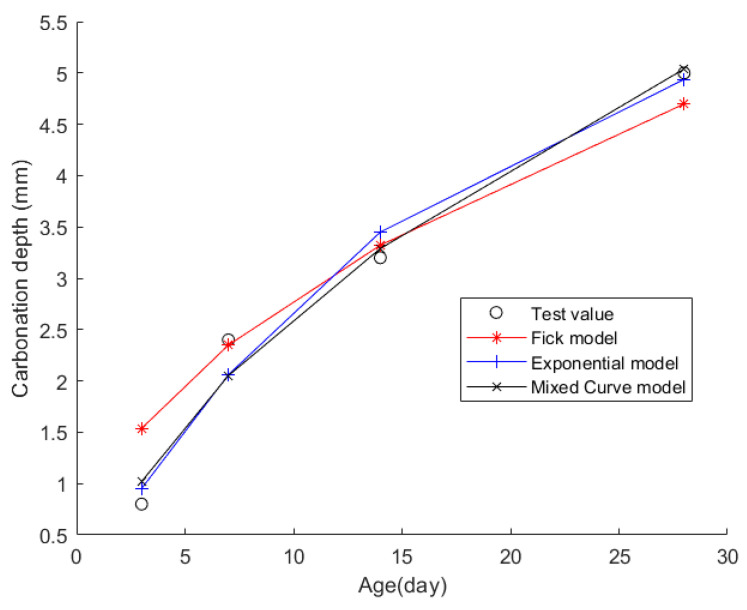
The fitting results of Case 3.

Then, the obtained data were put into the Fick, exponential, and mixed models for analyses. Figure 5 shows the final fitting curve. Table 9 shows the model’s average fitting error, standard deviation, and coefficient of determination ($R^{2}$). The specific fitting equations for the three models are as follows:

(24) $$z(t)=0.8878\sqrt{t}$$

(25) $$z(t)=6.0251-6.1031\cdot e^{-t/16.2279}$$

(26) $$z(t)=1.1291\sqrt{t}-1.1847\cdot \frac{\sqrt{t}}{\sqrt{t}+1}$$

Case 4: In citation [41], 12 concrete cube test blocks, each with a side length of 100 mm, were cast. Following standard curing for 26 days, the test blocks underwent drying at a constant temperature of 60 °C. Once they reached a curing age of 28 days, wax was applied to seal the test blocks around all sides except for two opposing ones. Subsequently, they were placed in a standard carbonation box for carbonation testing. The temperature, humidity, and CO_2_ concentration within the carbonation chamber all meet the requirements specified in the standard “Test Scheme for Long-term Performance and Durability of Ordinary Concrete” (GB/T 50082-2009) [48]. The CD of the concrete was measured at 3 days, 7 days, 14 days, and 28 days. The material composition of the concrete is outlined in Table 10, and the CD data at each time point are presented in Table 11.

**Figure 6:**
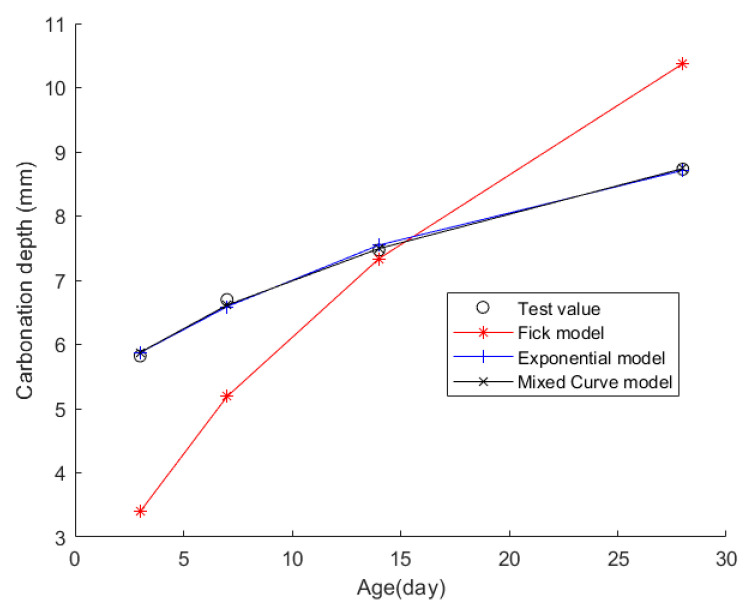
The fitting results of Case 4.

Then, the obtained data were put into the Fick, exponential, and mixed models for analyses. Figure 6 shows the final fitting curve. Table 12 shows the model’s average fitting error, standard deviation, and coefficient of determination ($R^{2}$). The specific fitting equations for the three models are as follows:

(27) $$z(t)=1.9606\sqrt{t}$$

(28) $$z(t)=9.897-4.6611\cdot e^{-t/20.437}$$

(29) $$z(t)=0.8046\sqrt{t}+5.6756\cdot \frac{\sqrt{t}}{\sqrt{t}+1}$$

Case 5: In citation [42], C30 cubic specimens measuring 100 × 100 × 100 mm were cast, demolded, and subsequently placed in a standard curing chamber for a period of 28 days. After this curing phase, the specimens were removed and subjected to a 48-hour drying process in an oven at 60 °C to halt hydration. Following this, the specimens were coated with wax for sealing and then placed in a concrete carbonation chamber for accelerated carbonation testing. The specimens were retrieved at various carbonation ages of 3d, 7d, 14d, and 28d to measure their CD. The experiment employed P·O 42.5 ordinary Portland cement as the primary cementitious material, complemented by a continuously graded limestone gravel ranging from 5 to 25 mm in size (with an apparent density of 2690 kg/m^3^, bulk density of 1470 kg/m^3^, crushing value of 14%, void ratio of 45%, and mud content of 0.2%). As fine aggregate, granite-based manufactured sand containing black mica was used (featuring an apparent density of 2670 kg/m^3^, stone dust content of 12.2%, mica content of 11.8%, water absorption rate of 2.2%, and fineness modulus of 3.1). To further enhance the mixture, a polycarboxylate superplasticizer with a water-reducing rate of 22.3% and a solids content of 20.0% was added, along with an admixture to regulate the workability and compactness of the concrete, boasting a solids content of 10.0%. Table 13 outlines the concrete material composition, while Table 14 displays the CD data recorded at each time point.

**Figure 7:**
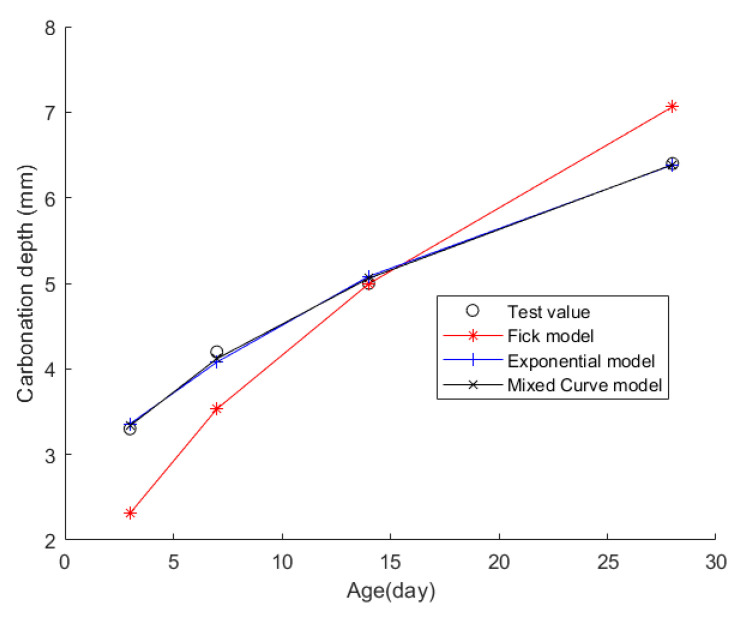
The fitting results of Case 5.

Then, the obtained data were put into the Fick, exponential, and mixed models for analyses. Figure 7 shows the final fitting curve. Table 15 shows the model’s average fitting error, standard deviation, and coefficient of determination ($R^{2}$). The specific fitting equations for the three models are as follows:

(30) $$z(t)=1.3347\sqrt{t}$$

(31) $$z(t)=7.9998-5.2683\cdot e^{-t/23.7058}$$

(32) $$z(t)=0.8577\sqrt{t}+2.3417\cdot \frac{\sqrt{t}}{\sqrt{t}+1}$$

Case 6: In citation [43], C25 prismatic specimens with dimensions of 100×100×400 mm were cast and subjected to accelerated carbonation testing in a concrete carbonation test chamber in accordance with the standard “Standard Test Methods for Long-term Performance and Durability of Ordinary Concrete” GB/T 50082-2009. The carbonation environment within the chamber was maintained at a temperature of (20 ± 2) °C, relative humidity of (70 ± 5)%, and a CO_2_ concentration of (20 ± 3)%. After carbonation ages of 3, 7, 14, 28, and 56 days, the specimens were retrieved to measure their CDs. The concrete mix design employed P·O 42.5 ordinary Portland cement. The fine aggregate was a blend of river sand and iron tailings sand in a mass ratio of 9:1, with an apparent density of 2659 kg/m^3^, a bulk density of 1548 kg/m^3^, a fineness modulus of 2.94, and a void ratio of 41%. The coarse aggregate was a continuous gradation from 5 mm to 20 mm, achieved by mixing iron tailings stones of 5 mm to 10 mm continuous grain size and 10 mm to 20 mm single-grain size in a mass ratio of 1:4. This coarse aggregate had an apparent density of 2695 kg/m^3^, a bulk density of 1576 kg/m^3^, a mud content of 2.6%, a void ratio of 39%, and a crushing index of 8.2%. The mineral admixtures included S95 grade slag powder (with an apparent density of 2910 kg/m^3^ and a moisture content of 0.44%), Class II fly ash (with an apparent density of 2240 kg/m^3^, a fineness of 19%, a water requirement of 104%, and a moisture content of 0.06%), and iron tailings powder (with an apparent density of 2960 kg/m^3^ and a specific surface area of 468 m^2^/kg). Additionally, polycarboxylate-based superplasticizer was added as an admixture, with a water-reducing rate of 25% and a solid content of 40%. Table 16 presents the material composition of the concrete, while Table 17 displays the CD data at each time point.

**Figure 8:**
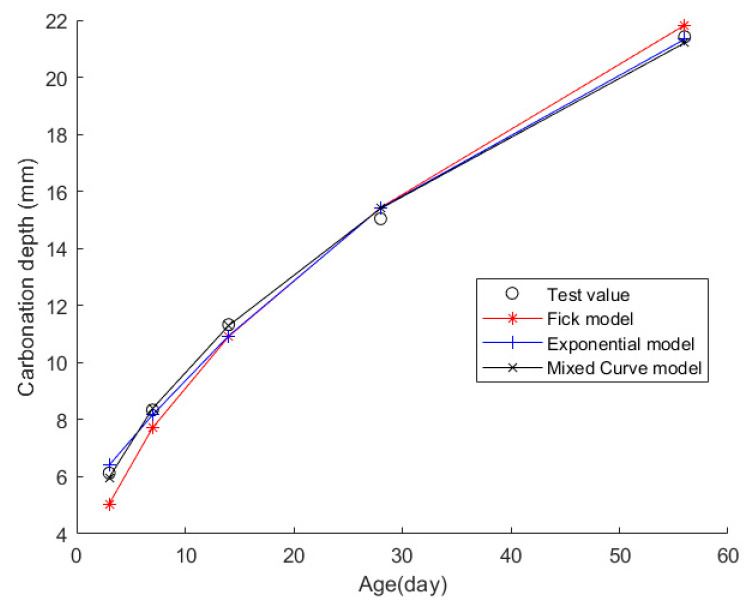
The fitting results of Case 6.

Then, the obtained data were put into the Fick, exponential, and mixed models for analyses. Figure 8 shows the final fitting curve. Table 18 shows the model’s average fitting error, standard deviation, and coefficient of determination ($R^{2}$). The specific fitting equations for the three models are as follows:

(33) $$z(t)=2.9162\sqrt{t}$$

(34) $$z(t)=29.0566-24.0750\cdot e^{-t/49.2683}$$

(35) $$z(t)=2.6481\sqrt{t}+1.6646\cdot \frac{\sqrt{t}}{\sqrt{t}+1}$$

Case 7: In citation [44], prismatic specimens of 100 mm × 100 mm × 300 mm were cast. Following the specifications outlined in the “Standard for Test Methods of Long-Term Performance and Durability of Ordinary Concrete” (GB/T50082-2009), a rapid carbonation method was employed. The test blocks were placed in a concrete carbonation test chamber for accelerated carbonation testing. Within the chamber, the concentration of carbon dioxide was maintained at (20 ± 3)%, humidity at (70 ± 5)%, and temperature at (20 ± 2) °C. The specimens were removed at intervals of 7, 14, 28, and 56 days for CD measurements. This experiment utilized a cementitious material consisting of ordinary Portland cement (with a strength grade of P·O 42.5R) mixed with fly ash having a fineness of 12 and a water requirement ratio of 93%. Natural crushed stone was used, along with fine aggregates composed of river sand (with an apparent surface area of 2689 kg/m^3^, a loose bulk density of 1655 kg/m^3^, a water absorption rate of 0.5%, and a mud content of 3.1%) and iron tailings sand (with an apparent surface area of 2745 kg/m^3^, a loose bulk density of 1824 kg/m^3^, a void ratio of 8.7%, a water absorption rate of 2.9%, and a mud content of 19.53%). Additionally, a naphthalene-based superplasticizer with a water-reducing rate of 20% was incorporated. Table 19 details the concrete’s material composition, while Table 20 presents the CD data for each time point.

**Figure 9:**
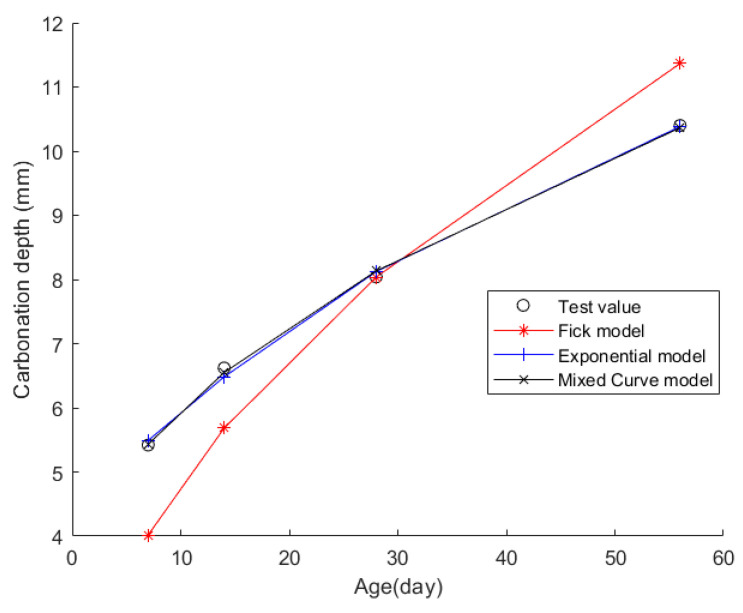
The fitting results of Case 7.

Then, the obtained data were put into the Fick, exponential, and mixed models for analyses. Figure 9 shows the final fitting curve. Table 21 shows the model’s average fitting error, standard deviation, and coefficient of determination ($R^{2}$). The specific fitting equations for the three models are as follows:

(36) $$z(t)=1.5189\sqrt{t}$$

(37) $$z(t)=13.8148-9.4456\cdot e^{-t/55.2963}$$

(38) $$z(t)=1.0198\sqrt{t}+3.2447\cdot \frac{\sqrt{t}}{\sqrt{t}+1}$$

Case 8: In citation [45], researchers cast standard-sized concrete blocks and put them in a chamber for quick carbonation tests, adhering to the “Standard for Test Methods of Long-Term Performance and Durability of Ordinary Concrete” (GB/T 50082-2009). They took out the blocks on days 3, 7, 14, 28, and 56 after carbonation to precisely gauge their carbonization depth. This study used P·O 42.5 cement, fly ash, natural and artificial sand, crushed stone, and other additives. Concrete composition details are in Table 22, while CDs are logged in Table 23.

**Figure 10:**
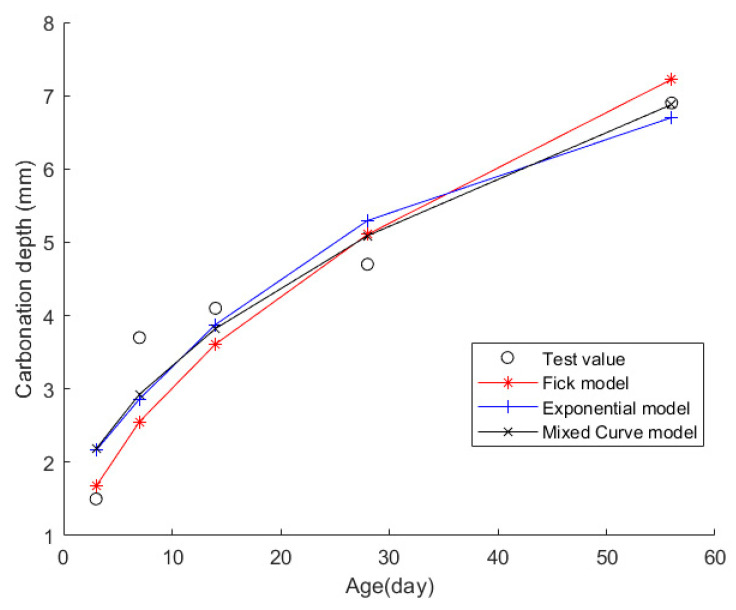
The fitting results of Case 8.

Then, the obtained data were put into the Fick, exponential, and mixed models for analyses. Figure 10 shows the final fitting curve. Table 24 shows the model’s average fitting error, standard deviation, and coefficient of determination ($R^{2}$). The specific fitting equations for the three models are as follows:

(39) $$z(t)=0.9651\sqrt{t}$$

(40) $$z(t)=7.5522-5.9846\cdot e^{-t/28.7005}$$

(41) $$z(t)=0.8167\sqrt{t}+0.9210\cdot \frac{\sqrt{t}}{\sqrt{t}+1}$$

Case 9: In citation [46], ten sets of prismatic specimens with dimensions of 100 mm × 100 mm × 400 mm were cast. These standard specimens were cured in a standard curing chamber for 28 days before being placed in a drying oven set at 60 °C for continuous drying for 48 h. Following the drying process, the specimens underwent wax sealing treatment and were then placed in a carbonation chamber where the CO_2_ concentration was maintained at (20 ± 3)%; the temperature was controlled at (20 ± 2) °C, and the relative humidity was regulated at (70 ± 5)%. The specimens were retrieved at 3d, 7d, 14d, and 28d intervals during the carbonation process to measure their CD. The cementitious material system employed in this experiment consisted primarily of P.O 42.5 ordinary Portland cement (with a specific surface area of 355 m^2^/kg), fly ash, and silica fume. For reinforcement, 18 mm-long basalt fibers were selected, featuring a density of 2.5 g/cm^3^ and an elastic modulus ranging from 91 to 110 GPa. To optimize the concrete’s performance, a polycarboxylate liquid water-reducing agent was incorporated at a dosage of 0.5% by the total mass of cementitious materials, offering a high water-reducing rate of 37%. Regarding fine aggregate, medium sand with a fineness modulus of 2.6 was chosen. This sand exhibits an apparent density of 2.610 g/cm^3^, a bulk density of 1.630 g/cm^3^, a moisture content controlled below 0.8%, and a silt content of less than 1.9%. As for coarse aggregate, crushed stone with a particle size ranging from 5 to 25 mm was utilized, possessing an apparent density of 2.753 g/cm^3^, a bulk density of 1.634 g/cm^3^, a moisture content of 0.15%, and a silt content of 0.4%. Table 25 and Table 26, respectively, showcase the detailed material composition of the concrete and the recorded CD data at each specified time point.

**Figure 11:**
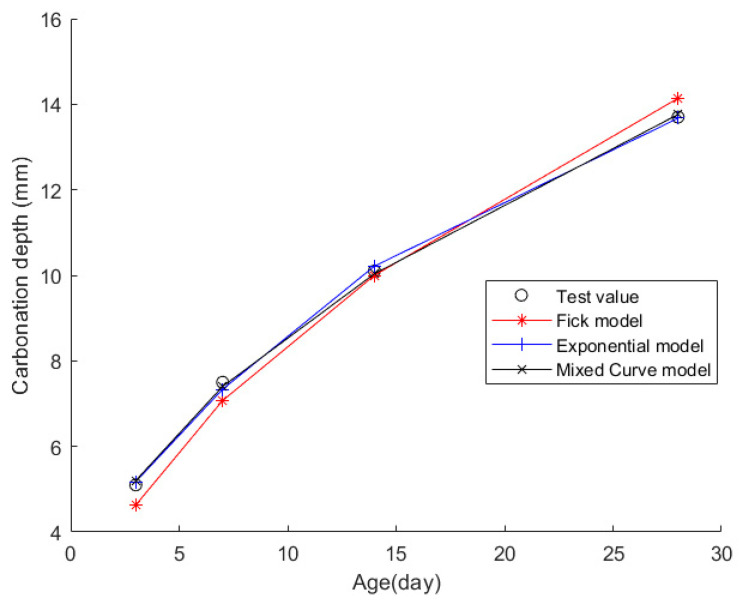
The fitting results of Case 9.

Then, the obtained data were put into the Fick, exponential, and mixed models for analyses. Figure 11 shows the final fitting curve. Table 27 shows the model’s average fitting error, standard deviation, and coefficient of determination ($R^{2}$). The specific fitting equations for the three models are as follows:

(42) $$z(t)=2.6723\sqrt{t}$$

(43) $$z(t)=17.0893-13.8396\cdot e^{-t/20.0009}$$

(44) $$z(t)=2.4048\sqrt{t}+1.3136\cdot \frac{\sqrt{t}}{\sqrt{t}+1}$$

Case 10: In citation [47], a carbonation test was conducted in accordance with the “Standard Test Methods for Long-Term Performance and Durability of Ordinary Concrete”. The test specimens measured 100 mm × 100 mm × 100 mm and were subjected to carbonation testing after being cured for 7 days. The specimens were retrieved, and their CDs were measured at 3 days, 7 days, 14 days, 28 days, and 56 days of carbonation. The raw materials used in this project comprised P.O42.5 grade cement with 3-day and 28-day compressive strengths reaching 31.5 MPa and 48.0 MPa respectively; Class F, Grade I fly ash (FA) featuring a water requirement ratio of 93% and a fineness of 7.3% (sieve residue on a 45 μm square hole sieve); S95 grade ground granulated blast furnace slag (KF) with a specific surface area of 425 m^2^/kg, maintaining a fluidity ratio of 100%, and an excellent 28-day mortar activity index of 104%; fine aggregate in the form of river sand with a fineness modulus of 2.81 and strict control of mud content below 1.6%; coarse aggregate composed of crushed stone with a crushing value of 15.1% and a flaky and elongated particle content of 4.9%, employing a two-stage grading of 5~20 mm, with a blending ratio of 4.75~9.5 mm to 9.5~19.0 mm particles at 2:8; and polycarboxylate superplasticizer as an admixture, boasting a water-reducing efficiency of 27%. Table 28 presents the concrete material composition, while Table 29 displays the CD data at each time point.

**Figure 12:**
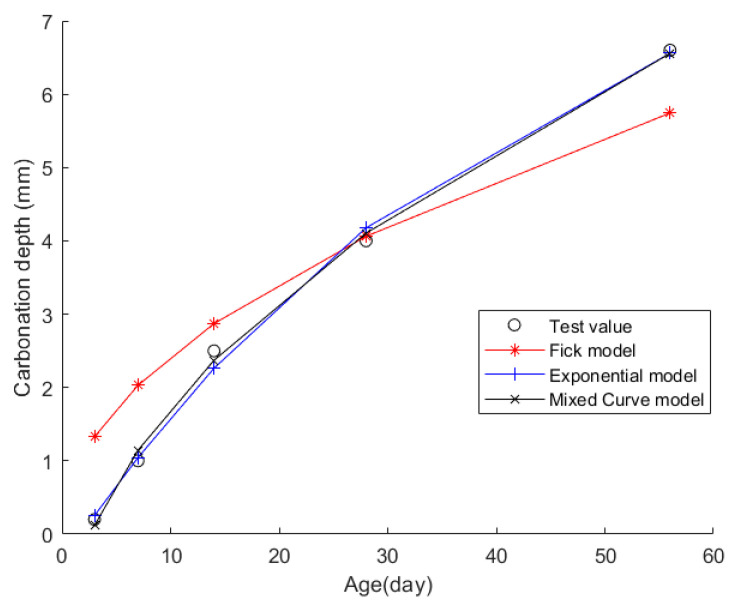
The fitting results of Case 10.

Then, the obtained data were put into the Fick, exponential, and mixed models for analyses. Figure 12 shows the final fitting curve. Table 30 shows the model’s average fitting error, standard deviation, and coefficient of determination ($R^{2}$). The specific fitting equations for the three models are as follows:

(45) $$z(t)=0.7676\sqrt{t}$$

(46) $$z(t)=9.1571-9.5449\cdot e^{-t/43.0316}$$

(47) $$z(t)=1.1189\sqrt{t}-2.1803\cdot \frac{\sqrt{t}}{\sqrt{t}+1}$$

Through a comprehensive analysis of the errors and coefficient of determination across the ten case studies, it is discernible that the novel model outperforms both the Fick and exponential models, exhibiting a diminished mean fitting error and a reduced standard deviation in its assessments. Additionally, the determination coefficients of the new model surpass those of the Fick and exponential models, underscoring the superior fitting quality of the new model. For example, compared to the second-best model, the new model exhibits a reduction of 59% and 50% in average fitting error and standard deviation in Case 1, 31% and 27% in Case 4, 33% and 32% in Case 5, and 37% and 29% in Case 6. These results demonstrate that this new model significantly enhances fitting accuracy. Upon meticulous examination of Figure 3, Figure 4, Figure 5, Figure 6, Figure 7, Figure 8, Figure 9, Figure 10, Figure 11 and Figure 12, the fitting curve of the novel model is observed to be more closely aligned with the experimental data points, indicating the model’s heightened precision in replicating the empirical observations. To sum up, the new model demonstrates superior data fitting capabilities relative to the Fick and exponential models, rendering it more precise and dependable in assessing concrete CD.

## 4. Conclusions

This article introduces a novel mathematical curve model for assessing the CD of concrete. By fitting mathematical curves using limited initial experimental data, this model offers a more accurate evaluation of concrete CD. The validity of this new model was confirmed through validation with various experimental data sets. The calculated results lead to the following key conclusions:

- The new model introduced in this article combines elements of the Fick and hyperbolic models. Unlike the index model, which may prematurely reach its limit value, the new model offers a more precise fit for the later stages of concrete carbonation;
- Compared to the Fick and exponential models, the new model introduced in this paper shows reduced fitting errors and higher $R^{2}$, indicating superior fitting precision. Unlike the exponential model, which necessitates fitting three unknown parameters, this new model only needs the fitting of two unknown parameters. This reduced parameter requirement makes this new model more efficient for practical engineering applications, requiring less data for fitting and easier implementation.

This article primarily focuses on the relationship between the CD of concrete and time without extensively addressing other factors that may influence this depth. The newly proposed model does not simultaneously account for multiple influencing variables about concrete CD. However, in contrast to multi-factor models, this new model requires only limited experimental data to derive the mathematical curve equation for evaluation. In scenarios where experimental data are scarce, this model can be integrated with machine learning techniques to simulate and generate additional data for training purposes, thereby enhancing the generalization capabilities of the machine learning models. The validation of the model presented in this article is based on a subset of the available data, indicating a need for further research to confirm that the model is fitting, evaluate capabilities for concrete, and optimize the fitting conditions. Additionally, this model may be relevant for the property parameters of other materials, and future experiments could be conducted to assess the model’s applicability in those contexts.

## Figures and Tables

**Table 1:** The material composition of the fly ash concrete test block in Case 1 (kg/m^3^).

Cement	Sand	Spall	Water	Fly Ash	Water–Binder Ratio
156	695	1135	175	234	0.45

**Table 2:** CD of concrete specimens at different ages in Case 1 (mm).

Age	0d	3d	7d	14d	28d
CD	1.4	6.2	9.4	11.2	16.6

**Table 3:** The fitting mean errors, standard deviations, and coefficient of determination from the three models in Case 1.

Index	Fick Model	Exponential Model	Mixed-Curve Model
Fitting mean error $\bar{\delta }$	0.8007	0.8130	0.3313
Fitted standard deviation σ	1.4056	1.3674	0.6854
Coefficient of determination ($R^{2}$)	0.9692	0.9709	0.9927

**Table 4:** The material composition of the lime powder concrete test block in Case 2 (kg/m^3^).

Cement	Sand	Spall	Water	Limestone Powder	Admixture
306	850	1030	170	34	5.1

**Table 5:** CD of concrete specimens at different ages in Case 2 (mm).

Age	14d	28d	60d	90d
CD	0	1.1	1.9	2.8

**Table 6:** The fitting mean errors, standard deviations, and coefficient of determination from the three models in Case 2.

Index	Fick Model	Exponential Model	Mixed-Curve Model
Fitting mean error $\bar{\delta }$	0.3972	0.1445	0.1201
Fitted standard deviation σ	1.0513	0.3086	0.2952
Coefficient of determination ($R^{2}$)	0.7399	0.9776	0.9795

**Table 7:** The material composition of the concrete test block in Case 3 (kg/m^3^).

Cement	Sand	Spall	W/C	Fly Ash
300	856	975	0.5	60

**Table 8:** CD of concrete specimens at different ages in Case 3 (mm).

Age	3d	7d	14d	28d
CD	0.8	2.4	3.2	5.0

**Table 9:** The fitting mean errors, standard deviations, and coefficient of determination from the three models in Case 3.

Index	Fick Model	Exponential Model	Mixed-Curve Model
Fitting mean error $\bar{\delta }$	0.3032	0.2008	0.1741
Fitted standard deviation σ	0.8081	0.4523	0.4233
Coefficient of determination ($R^{2}$)	0.9286	0.9776	0.9804

**Table 10:** The material composition of the concrete test block in Case 4 (kg/m^3^).

Cement	Sand	Spall	Water Reducing Agent	Fly Ash	Silica Fume	Water	W/C
380	655	983	4.9	40	74	101.1	0.38

**Table 11:** CD of concrete specimens at different ages in Case 4 (mm).

Age	3d	7d	14d	28d
CD	5.82	6.70	7.47	8.73

**Table 12:** The fitting mean errors, standard deviations, and coefficient of determination from the three models in Case 4.

Index	Fick Model	Exponential Model	Mixed-Curve Model
Fitting mean error $\bar{\delta }$	1.4289	0.0648	0.0444
Fitted standard deviation σ	3.2996	0.1471	0.1079
Coefficient of determination ($R^{2}$)	−1.3841	0.9953	0.9974

**Table 13:** The material composition of the concrete test block in Case 5 (kg/m^3^).

Cement	Sand	Spall	Water Reducing Agent	Regulator	Water
400	818	1038	6.8	40	165

**Table 14:** CD of concrete specimens at different ages in Case 5 (mm).

Age	3d	7d	14d	28d
CD	3.7	4.8	5.1	7.6

**Table 15:** The fitting mean errors, standard deviations, and coefficient of determination from the three models in Case 5.

Index	Fick Model	Exponential Model	Mixed-Curve Model
Fitting mean error $\bar{\delta }$	0.5814	0.0694	0.0468
Fitted standard deviation σ	1.3649	0.1580	0.1069
Coefficient of determination ($R^{2}$)	0.6409	0.9952	0.9978

**Table 16:** The material composition of the concrete test block in Case 6 (kg/m^3^).

Cement	Water	Sand-Aggregate	Water–Binder Ratio	Iron Tailings Powder	Fly Ash	Slag Powder	Water Reducing Agent	Spall	Sand
141	180	43.5	0.57	31	47	94	1.85	1055	812

**Table 17:** CD of concrete specimens at different ages in Case 6 (mm).

Age	3d	7d	14d	28d	56d
CD	6.13	8.34	11.33	15.04	21.42

**Table 18:** The fitting mean errors, standard deviations, and coefficient of determination from the three models in Case 6.

Index	Fick Model	Exponential Model	Mixed-Curve Model
Fitting mean error $\bar{\delta }$	0.5832	0.2609	0.1642
Fitted standard deviation σ	1.0111	0.4527	0.3198
Coefficient of determination ($R^{2}$)	0.9859	0.9971	0.9986

**Table 19:** The material composition of the concrete test block in Case 7 (kg/m^3^).

Cement	Water	Sand	Spall	Iron Tailings Sand	Fly Ash	Water Reducing Agent
440	220	459.2	1103	196.8	110	2.2

**Table 20:** CD of concrete specimens at different ages in Case 7 (mm).

Age	7d	14d	28d	56d
CD	5.42	6.62	8.04	10.40

**Table 21:** The fitting mean errors, standard deviations, and coefficient of determination from the three models in Case 7.

Index	Fick Model	Exponential Model	Mixed-Curve Model
Fitting mean error $\bar{\delta }$	0.8269	0.0771	0.0518
Fitted standard deviation σ	1.9430	0.1769	0.1201
Coefficient of determination ($R^{2}$)	0.7253	0.9977	0.9990

**Table 22:** The material composition of the concrete test block in Case 8 (kg/m^3^).

Cement	Water	Artificial Sand	Natural Sand	5–10 Stone	10–20 Stone	Fly Ash	Proportion of Additives (%)
317	175	244	569	250	779	56	2.1

**Table 23:** CD of concrete specimens at different ages in Case 8 (mm).

Age	3d	7d	14d	28d	56d
CD	1.5	3.7	4.1	4.7	6.9

**Table 24:** The fitting mean errors, standard deviations, and coefficient of determination from the three models in Case 8.

Index	Fick Model	Exponential Model	Mixed-Curve Model
Fitting mean error $\bar{\delta }$	0.5073	0.5031	0.4281
Fitted standard deviation σ	0.9624	0.8896	0.8028
Coefficient of determination ($R^{2}$)	0.8772	0.8951	0.9146

**Table 25:** The material composition of the concrete test block in Case 9 (kg/m^3^).

Cement	Water	Fine Aggregate	Coarse Aggregate	Water Reducing Agent (%)
441	150	511.68	1314.18	0.5%

**Table 26:** CD of concrete specimens at different ages in Case 9 (mm).

Age	3d	7d	14d	28d
CD	5.1	7.5	10.1	13.7

**Table 27:** The fitting mean errors, standard deviations, and coefficient of determination from the three models in Case 9.

Index	Fick Model	Exponential Model	Mixed-Curve Model
Fitting mean error $\bar{\delta }$	0.3607	0.0954	0.0824
Fitted standard deviation σ	0.7818	0.2164	0.1692
Coefficient of determination ($R^{2}$)	0.9850	0.9989	0.9993

**Table 28:** The material composition of the concrete test block in Case 10 (kg/m^3^).

Cement	Water	Fly Ash	Slag Powder	Sand	Stone	Water–Binder Ratio	Water Reducing Agent Dosage
329	146	94	47	743	1114	0.31	1.25%

**Table 29:** CD of concrete specimens at different ages in Case 10 (mm).

Age	3d	7d	14d	28d	56d
CD	0.2	1.0	2.5	4.0	6.6

**Table 30:** The fitting mean errors, standard deviations, and coefficient of determination from the three models in Case 10.

Index	Fick Model	Exponential Model	Mixed-Curve Model
Fitting mean error $\bar{\delta }$	0.6900	0.1111	0.0998
Fitted standard deviation σ	1.2675	0.2173	0.1680
Coefficient of determination ($R^{2}$)	0.8762	0.9964	0.9978

## Data Availability

The original contributions presented in the study are included in the article, further inquiries can be directed to the corresponding authors.

## References

[1] Liu L.L. (2021). Study on the Absorption of CO_2_ by Fresh Cement Slurry and Its Reverse Mechanism. Ph.D. Thesis.

[2] Wang X., Yang Q., Peng X., Qin F. (2024). A Review of Concrete Carbonation Depth Evaluation Models. Coatings.

[3] Felix E.F., Carrazedo R., Possan E. (2021). Carbonation model for fly ash concrete based on artificial neural network: Development and parametric analysis. Constr. Build. Mater..

[4] Ventura A., Ta V.L., Kiessé T.S., Bonnet S. (2021). Design of concrete: Setting a new basis for improving both durability and environmental performance. J. Ind. Ecol..

[5] Medvedev V., Pustovgar A. (2023). A Review of Concrete Carbonation and Approaches to Its Research under Irradiation. Buildings.

[6] Chen G., Lv Y., Zhang Y., Yang M. (2021). Carbonation depth predictions in concrete structures under changing climate condition in China. Eng. Fail. Anal..

[7] Peng X., Shi F., Yang J., Yang Q., Wang H., Zhang J. (2023). Modification of construction waste derived recycled aggregate via CO_2_ curing to enhance corrosive freeze-thaw durability of concrete. J. Clean. Prod..

[8] Malami S.I., Akpinar P., Lawan M.M. Preliminary investigation of carbonation problem progress in concrete buildings of north Cyprus. Proceedings of the MATEC Web of Conferences.

[9] Jiang C., Gu X., Huang Q., Zhang W. (2018). Carbonation depth predictions in concrete bridges under changing climate conditions and increasing traffic loads. Cem. Concr. Compos..

[10] Forsdyke J.C., Lees J.M. (2023). Model fitting to concrete carbonation data with non-zero initial carbonation depth. Mater. Struct..

[11] Fuhaid A.F.A., Niaz A. (2022). Carbonation and corrosion problems in reinforced concrete structures. Buildings.

[12] Jeong H., Jung B.J., Kim J.H., Seo S.Y., Kim K.S. (2022). Development and assessment of Nile blue-immobilized pH sensor to monitor the early stage of concrete carbonation. J. Build. Eng..

[13] Majlesi A., Koodiani H.K., de Rincon O.T., Montoya A., Millano V., Torres-Acosta A.A., Troconis B.C.R. (2023). Artificial neural network model to estimate the long-term carbonation depth of concrete exposed to natural environments. J. Build. Eng..

[14] Tran V.Q. (2022). Using Artificial neural network containing two hidden layers for predicting carbonation depth of concrete. CIGOS 2021, Emerging Technologies and Applications for Green Infrastructure: Proceedings of the 6th International Conference on Geotechnics, Civil Engineering and Structures.

[15] Concha N.C. (2024). A robust carbonation depth model in recycled aggregate concrete (RAC) using neural network. Expert Systems with Applications. Expert Syst. Appl..

[16] Akpinar P., Uwanuakwa I. (2020). Investigation of the parameters influencing progress of concrete carbonation depth by using artificial neural networks. Mater. Construcción.

[17] Zhang K., Zhang K., Bao R., Liu X. (2023). A framework for predicting the carbonation depth of concrete incorporating fly ash based on a least squares support vector machine and metaheuristic algorithms. J. Build. Eng..

[18] Huang S., Wang L., Li Z., Su D., Luo Q. (2024). Machine learning-based prediction model for CO_2_-induced corrosion on oil well cement under high-pressure and high-temperature condition. Constr. Build. Mater..

[19] Çevik A., Kurtoğlu A.E., Bilgehan M., Gülşan M.E., Albegmprli H.M. (2015). Support vector machines in structural engineering: A review. J. Civ. Eng. Manag..

[20] Li Z., He H., Zhao S. Research on support vector machine’s prediction of concrete carbonization. Proceedings of the 2008 International Seminar on Business and Information Management.

[21] Xiang R. Prediction of concrete carbonation depth based on support vector regression. Proceedings of the 2009 Third International Symposium on Intelligent Information Technology Application.

[22] Londhe S., Kulkarni P., Dixit P., Silva A., Neves R., De Brito J. (2022). Tree based approaches for predicting concrete carbonation coefficient. Appl. Sci..

[23] Wang J., Zhang Z.Q., Liu X.M., Shao Y., Liu X.Y., Wang H.M. (2024). Prediction and interpretation of concrete corrosion induced by carbon dioxide using machine learning. Corros. Sci..

[24] Moghaddas S.A., Nekoei M., Golafshani E.M., Nehdi M., Arashpour M. (2022). Modeling carbonation depth of recycled aggregate concrete using novel automatic regression technique. J. Clean. Prod..

[25] Lee H., Lee H., Suraneni P. (2020). Evaluation of carbonation progress using AIJ model, FEM analysis, and machine learning algorithms. Constr. Build. Mater..

[26] Kumar A., Arora H.C., Kapoor N.R., Kontoni D.P.N., Kumar K., Jahangir H., Bhushan B. (2023). Practical applicable model for estimating the carbonation depth in fly-ash based concrete structures by utilizing adaptive neuro-fuzzy inference system. Comput. Concr..

[27] Koichi K. (1963). Durability of Iron Reinforced Concrete.

[28] Zhu A.M. (1992). Carbonization of Concrete and Durability of Reinforced Concrete. Concrete.

[29] Liu Z.Y. (2006). Research on Durability Testing and Life Prediction Method of Marine Concrete Based on Environment. Ph.D. Thesis.

[30] Niu D.T. (2003). Durability and Life Prediction of Concrete Structures.

[31] Xu L.P., Huang S.Y. (1991). Mathematical Model for Predicting Carbonation Depth in Concrete. J. Shanghai Build. Mater. Inst..

[32] Gong L.S., Su M.Q., Wang H.L. (1985). Multi coefficient carbonation equation of concrete and its application. Concr. Reinf. Concr..

[33] Papadakis V.G., Vayenas C.G., Fardis M.N. (1991). Fundamental modeling and experimental investigation of concrete carbonation. Mater. J..

[34] Possan E., Andrade J., Dal Molin D., Ribeiro J. (2021). Model to estimate concrete carbonation depth and service life prediction. Hygrothermal Behaviour and Building Pathologies.

[35] Ekolu S.O. (2018). Model for practical prediction of natural carbonation in reinforced concrete: Part 1-formulation. Cem. Concr. Compos..

[36] Liang M.T., Lin S.M. (2003). Mathematical modeling and applications for concrete carbonation. J. Mar. Sci. Technol..

[37] Si X.Y., Hu W.H., Pan H.M. (2024). Anti carbonation performance and prediction model of mineral admixture concrete. Concrete.

[38] Deng S.F. (2023). Research on predicting the full life of concrete based on carbonation durability. Constr. Mach..

[39] Zhang J.Y., Gu S.Y., Li Q.F., Huang H.L. (2022). The influence of mineral admixtures on the carbonation performance of concrete. Jiangxi Build. Mater..

[40] Zhang C., Song C.Q., Yao J., Song X.Y., Shi P.C. (2023). The influence and mechanism analysis of Basf early strength agent on the durability performance of concrete. Technol. Innov. Appl..

[41] Ding Y.H., Guo S.Q., Zhang X.G., Xu P., Wu J., Zhang M.X. (2022). The influence of basalt fiber on the carbonation resistance of recycled concrete. J. Compos. Mater..

[42] Qin M.T. (2023). Preparation and Performance Study of Granite Mechanized Sand Concrete. Master’s Thesis.

[43] Liang X.G. (2022). Carbonization and Mix Proportion Optimization of C20~C30 Concrete Mixed with Iron Tailings Powder.

[44] Zheng W.B. (2022). Experimental Study on Carbonation Performance of Hybrid Fiber-Reinforced Iron Tailings Sand Concrete.

[45] Xue L.Y., Hao Q.J., Zhang W.K., Sun Q. (2021). Experimental study on the influence of different factors on the carbonation resistance of concrete. Cem. Eng..

[46] Wang S.L. (2021). Research on the Mechanical and Carbonization Properties of Mineral Admixtures and Basalt Fiber Reinforced Concrete.

[47] Xu X.H., Xie J.F., Xia W.H., Li B.X. (2021). The influence of mineral admixtures on the performance and microstructure of bridge tower concrete. China Harb. Constr..

[48] (2009). Standard for Test Methods of Long-Term Performance and Durability of Ordinary Concrete.

